# Improving quantitation accuracy in isobaric-labeling mass spectrometry experiments with spectral library searching and feature-based peptide-spectrum match filter

**DOI:** 10.1038/s41598-023-41124-2

**Published:** 2023-08-29

**Authors:** Tzu-Yun Kuo, Jen-Hung Wang, Yung-Wen Huang, Ting-Yi Sung, Ching-Tai Chen

**Affiliations:** 1 Department of Biochemical Science and Technology, National Taiwan University, Taipei, 10617 Taiwan; 2 Bioinformatics Program, Taiwan International Graduate Program, Institute of Statistical Science, Academia Sinica, Taipei, 11529 Taiwan; 3 Institute of Information Science, Academia Sinica, Taipei, 11529 Taiwan; 4 Institute of Biomedical Informatics, National Yang Ming Chiao Tung University, Taipei, 11221 Taiwan; 5 Department of Computer Science and Information Engineering, National Taiwan University, Taipei, 10617 Taiwan; 6 Department of Bioinformatics and Biomedical Engineering, Asia University, Taichung, 41354 Taiwan; 7 Center for Precision Health Research, Asia University, Taichung, 41354 Taiwan

**Keywords:** Bioinformatics, Proteomic analysis, Proteome informatics

## Abstract

Isobaric labeling relative quantitation is one of the dominating proteomic quantitation technologies. Traditional quantitation pipelines for isobaric-labeled mass spectrometry data are based on sequence database searching. In this study, we present a novel quantitation pipeline that integrates sequence database searching, spectral library searching, and a feature-based peptide-spectrum-match (PSM) filter using various spectral features for filtering. The combined database and spectral library searching results in larger quantitation coverage, and the filter removes PSMs with larger quantitation errors, retaining those with higher quantitation accuracy. Quantitation results show that the proposed pipeline can improve the overall quantitation accuracy at the PSM and protein levels. To our knowledge, this is the first study that utilizes spectral library searching to improve isobaric labeling-based quantitation. For users to conveniently perform the proposed pipeline, we have implemented the feature-based filter being executable on both Windows and Linux platforms; its executable files, user manual, and sample data sets are freely available at https://ms.iis.sinica.edu.tw/comics/Software_FPF.html. Furthermore, with the developed filter, the proposed pipeline is fully compatible with the Trans-Proteomic Pipeline.

## Introduction

Mass spectrometry (MS)-based proteomics has become a powerful technology for the identification and quantitation of protein mixtures in complex samples^1^. Isobaric labeling, a widely used protein quantitation technique, has the advantage of multiplexed and high-throughput capabilities which enables quantifying thousands of proteins from multiple samples in a single run^2^. TMT (Tandem Mass Tag)^3^ and iTRAQ (isobaric Tags for Relative and Absolute Quantitation)^4^ are the two most commonly used reagents. For example, a number of research teams from CPTAC^5, 6^ (Clinical Proteomic Tumor Analysis Consortium) use TMT in analyzing oncoproteomic data for cancer research.

Several existing isobaric labeling quantitation tools support identification results from a single search engine for quantitation; for instance, MaxQuant^7^ supports Andromeda^8^, and PatternLab^9^ supports Comet^10^. Some other tools support the results from multiple search engines, such as Libra, which is included in the Trans-Proteomic Pipeline (TPP)^11, 12^, Multi-Q 2^13^, and the commercial Proteome Discoverer; these tools in general have a larger coverage of quantifiable peptides and proteins. Notably, in recent years, spectral library searching that matches a query spectrum against a library of experimental reference spectra with known identifications has been emerging as a complementary approach to database searching. Spectral library searching utilizes the intensities of fragment ions for spectrum matching, thereby enhancing sensitivity and reducing search time^14–19^. This approach has been demonstrated to improve the identification of TMT-labeled peptides by sequence database searching^20^. Previous studies on spectral library searching have made efforts to identification, but systematic analysis of its impact on quantitation accuracy remains limited^21^. Specifically, the use of spectral library searching to improve quantitation accuracy of isobaric-labeled data requires further investigation.

In this study, we construct a pipeline that integrates sequence database searching, spectral library searching, and a spectrum filter to achieve better quantitation accuracy for isobaric-labeling MS experiments. First, sequence database searching is applied to the isobaric-labeled spectral files. A sample-specific spectral library is then constructed with database search results, which is used for spectral library searching. The combined results of database and spectral library searching contain more peptide-spectrum matches (PSMs) than using database searching alone for subsequent quantitation analysis. Next, we develop a software tool, the feature-based PSM filter (FPF), in an attempt to filter out PSMs with larger quantitation errors by examining various spectral features, such as peptide length, charge state, and average reporter ion intensity. Diverse features are considered because they can be significantly correlated with the accuracy of quantitation ratios according to Fischer and Renard’s study^22^. The identification results of combined database and spectral library searching are processed by FPF to remove PSMs with larger quantitation errors, and the resulting PSMs are used for quantitation, thereby improving quantitation accuracy. Our experiment results show that the PSMs removed by FPF have median AREs (average relative errors) of 0.407, 0.173, and 0.172 for three standard data sets, respectively, while the PSMs retained by FPF have significantly smaller median AREs of 0.083, 0.105, and 0.094. It is demonstrated that the proposed pipeline includes more PSMs with higher quantitation accuracy while removing a majority of PSMs with lower accuracy in general, resulting in improved quantitation performance compared to conventional quantitation workflow based on only sequence database searching. Noteworthily, the quantitation improvement can be achieved at the protein level, even though spectral library searching usually increases sensitivity at the PSM and peptide levels. For example, the proposed pipeline results in 8.3% and 52.9% increases in the number of more accurately quantified proteins, i.e., proteins with ARE smaller than 0.04 and 0.075 for two standard data sets, respectively, compared to the conventional approach. The study demonstrates that the integration of FPF and spectral library searching, initially developed to enhance the sensitivity of PSM identification, can lead to improved quantitation accuracy for isobaric labeling experiments. FPF, a lightweight and installation-free tool, is designed to be fully compatible with TPP and can be executed on both Windows and Linux platforms. Its source code is open and freely available to public.

## Materials and methods

### Standard proteomics data sets

Three data sets were downloaded from the public domain for this study. The first data set, denoted as DS-Schmidt, was downloaded from the ProteomeXchange Consortium^23^ with the identifier PXD003346^24^. The data set consisted of six samples with varying amounts of *B*. *henselae* peptide digests (from 0.5 μL up to 25 μL) mixed with 40 μL of HeLa S3 peptide digests. The mixtures of samples were intentionally designed so that PSM ratios of human proteins are closer to their ideal values and those of *B*. *henselae* proteins are less accurate because of the interference of co-eluting peptides. Thus, we took only human proteins to demonstrate the capability of FPF. Taking the intensity of the first channel as the denominator and those of the rest as numerators, we obtained five protein ratios with theoretical values of 1 for each human protein.

The second data set, named DS-NCI-7^25^, was downloaded from the CPTAC Data Portal^6^. It consisted of TMT-10 samples from seven different cancer cell lines. Channels one to three, four to six, and seven to nine are of proportions 1:1:0.5 from three biological replicates of the digested mixtures, respectively. The last channel is a pooled reference generated with equal amounts of proteins from each cell line. In this data set, the last channel was used as the denominator, leading to nine ratios with theoretical values of either 1 or 0.5.

The third data set, denoted as DS-Yang, was downloaded from the ProteomeXchange Consortium with the identifier PXD005486^26^. Thirteen proteins were spiked in with different concentrations ranging from 0 to 80 pmol in 10 *E. coli* lysates of 70 μg. We took the intensity of the first channel as the denominator and those of the rest as numerators, resulting in nine ratios with theoretical values of 1 for *E. coli* proteins and theoretical values ranging from 0.025 to 20 for spiked-in proteins.

For peptide and protein identifications, all data sets were searched with Comet and X!Tandem^27^, for which the databases and search parameters were identical to those described in the original papers, listed in Supplementary Tables S1−S2. Reporter ions of each spectrum were normalized according to the median intensity of each channel across all the identified spectra^13^. In this study, the DS-Schmidt data set was used to determine a set of filtering conditions through the analyses of quantitation errors and various spectral features. Based on the set of filtering conditions, all three data sets were used to evaluate the quantitation performance of the proposed workflow.

### Overview of the workflow

**Figure 1:**
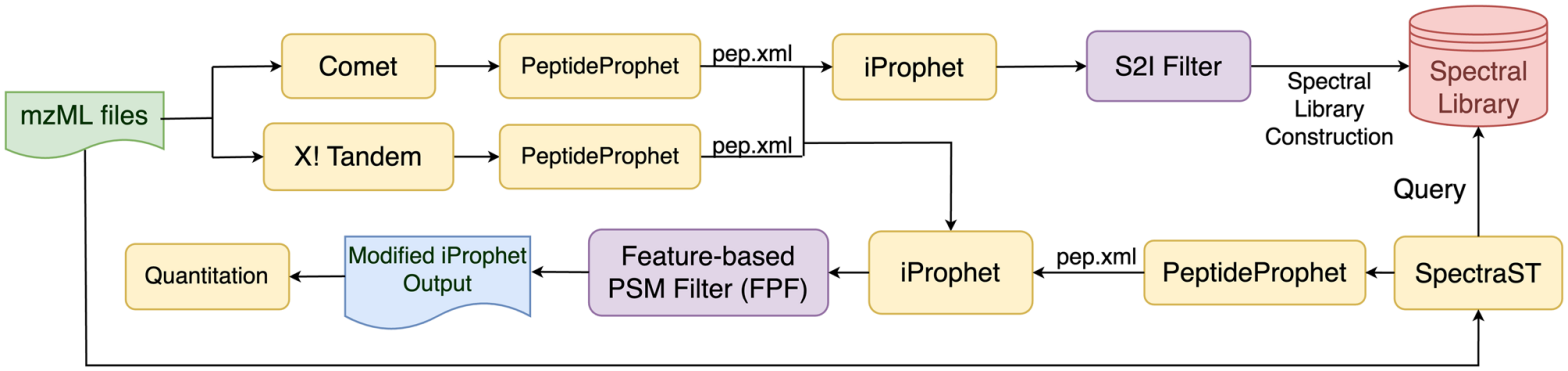
The workflow of spectral library-assisted isobaric labeling quantitation with feature-based PSM filter (FPF).

The workflow proposed in this study is briefly described as follows. Peptide identification via sequence database (DB) searching is performed on spectral files in the mzML/mzXML format and the identification results are used to build up a sample-specific spectral library (SL). Next, we perform SL searching on the entire data set, and the output is combined with the output from DB searching using statistical validation tools provided by TPP. The identification results based on DB searching are then compared with identification results based on the combined DB and SL searching, denoted as DB + SL searching, to distinguish the common and distinct PSMs between both approaches. For convenience, the PSMs exclusively belonging to the identification results of DB searching are termed DB-exclusive PSMs, and those exclusively belonging to the results of DB + SL searching are termed DB + SL-exclusive PSMs. Then only DB + SL-exclusive PSMs are processed by FPF to remove PSMs with larger quantitation errors. The remaining PSMs from DB + SL searching can lead to improved quantitation compared to the conventional approach using DB searching alone, because they contain more PSMs with relatively minor quantitation errors. A more detailed workflow is illustrated in Fig. 1.

### DB searching and SL construction

As illustrated in the upper half of Fig. 1, the mzML or mzXML files of an input data set are searched by Comet and X!Tandem, followed by PeptideProphet^28^ for statistical validation, and then both resulting pep.xml files are combined and validated by iProphet^29^ to obtain the results of DB searching, i.e., PSMs with a false discovery rate (FDR) of 1% at the peptide level (called valid PSMs in this paper). For all the valid PSMs, the signal-to-interference (S2I) measure is calculated as the abundance of a precursor and its isotopic clusters divided by the sum of all ion signals observed within the isolation window. To eliminate the PSMs affected by higher co-eluting interferences, an S2I filter removes PSMs with S2I values smaller than 0.7. The resulting PSMs are used to construct a sample-specific SL using SpectraST^16, 30^ with default parameters as listed in Supplementary Table S3.

### DB + SL searching and FPF

As illustrated in the lower half of Fig. 1, SpectraST is used to re-search the input mzML/mzXML files against the SL using parameters listed in Supplementary Table S4, followed by PeptideProphet for statistical validation. The resulting pepXML file and the two pepXML files from database searching (based on Comet and X!Tandem) are processed by iProphet, producing an ipro.pep.xml file consisting of PSMs identified by the three search engines, i.e., by DB + SL searching, with an FDR < 1% at the peptide level. The file is compared to the iProphet output based on DB searching alone to obtain DB-exclusive PSMs and DB + SL-exclusive PSMs. FPF is then applied on DB + SL-exclusive PSMs to filter out PSMs with larger quantitation errors, retaining PSMs with better quality for quantitation. Because we regard DB search results as the baseline for quantitation analysis, applying FPF on only DB + SL-exclusive PSMs allows us to perform a direct comparison between the proposed pipeline and the conventional pipeline based on DB searching alone. The output of FPF is an ipro.pep.xml file modified from the identification results of DB + SL searching that is fully compatible with the original iProphet format, facilitating a follow-up protein level identification analysis with ProteinProphet^31^ or Mayu^32^ and quantitation analysis with Libra or Multi-Q 2.

### FPF features

FPF takes into account the following features of PSMs for filtering: (1) precursor’s charge state, (2) precursor’s mass, (3) peptide length, (4) number of PTMs in a peptide, (5) PTM ratio, defined as the number of PTMs in a peptide divided by peptide length, (6) absolute mass difference, which is the absolute difference between the observed and theoretical peptide mass, (7) average reporter ion intensity, calculated from normalized reporter ion intensity of each channel, (8) F-value, (9) intra-protein distance (IProtDist), the distance between a PSM to all the other PSMs in the same protein in terms of PSM ratios, and (10) intra-peptide distance (IPepDist), the distance between a PSM to all the other PSMs assigned to the same peptide in terms of PSM ratios. When a PSM has missing reporter ion(s), FPF bypasses the filtering conditions of average reporter ion intensity, IProtDist, and IPepDist for this PSM.

IProtDist of PSM* j*, denoted as *IProtDist*_*j*_, is calculated as

$$IProtDist_{j} = \sqrt {\mathop \sum \limits_{{i{ } = 1}}^{n} \left( {\frac{{R_{i}^{j} - Avg\_Rest_{i} }}{{Avg_{i} }}} \right)^{2} }$$

where *R*_*i*_^*j*^ stands for the *i*^*th*^ ratio of PSM *j*; *Avg_Rest*_*i*_ stands for the average of the *i*^*th*^ ratio across all the PSMs except for PSM *j* within the protein; *Avg*_*i*_ stands for the average of the *i*^*th*^ ratio across all PSMs within the protein; *n* stands for the number of predefined ratios. *IProtDist*_*j*_ is zero if PSM *j* is the only PSM assigned to the protein.

The calculation of IPepDist is the same as IProtDist, except that *Avg_Rest*_*i*_ and *Avg*_*i*_ are calculated based on PSMs assigned to the same peptide instead of those assigned to the same protein. For single-hit peptides (peptides identified by only a single PSM), *Avg_Rest*_*i*_, and *Avg*_*i*_ cannot be calculated; thus, we merge all such peptides from a protein as a pseudo-peptide, for which *Avg_Rest*_*i*_, and *Avg*_*i*_ are calculated. Similarly, *IPepDist*_*j*_ is zero if PSM *j* is the only PSM assigned to the pseudo-peptide.

In addition, FPF also examines features exclusive to the SpectraST output file, including (1) absolute precursor m/z difference, (2) dot product, (3) delta score, (4) number of hits, (5) mean of the dot products of the hits, denoted as hit_mean, and (6) standard deviation of the dot products of the hits, denoted as hit_std. For some PSMs without such features reported in the iProphet file, FPF directly ignores the filtering conditions regarding these features.

### Filtering conditions for FPF

For DS-Schmidt, we collected DB + SL-exclusive PSMs and calculated their AREs, which is defined as the average of |*x*_*i*_-*y*_*i*_|/*y*_*i*_ over all the predefined ratios in a PSM, where *x*_*i*_ is the observed ratio and *y*_*i*_ is the theoretical ratio. We regarded the 25% PSMs with the top AREs in a data set as PSMs with larger quantitation errors, which were expected to be filtered out.

Filtering conditions for FPF are determined under the rationale that the eliminated PSMs of a certain condition should include as many PSMs with larger quantitation errors as possible, while the majority of PSMs of better quantitation accuracy are preserved. In this study, we used the DS-Schmidt data set to analyze the distribution of PSMs with larger quantitation errors on each single feature described above for the sake of generating reasonable filtering conditions for DB + SL-exclusive PSMs. Such conditions were then applied on DS-Schmidt, DS-Yang, and DS-NCI-7 data sets for quantitation analysis. Please note that the default parameters of FPF conditions were set based on the analysis of DS-Schmidt, but these parameters are adjustable in the tool by users.

### Evaluation of quantitation results

We used ARE and area under the curve of PSM coverage vs. PSM ARE, denoted as AUC, to evaluate quantitation results at the PSM level. The former is described in the previous section; the latter is calculated by the area under the curve for which the x-axis denotes the PSM ARE, and the y-axis denotes the coverage of PSM within the ARE. A large AUC indicates larger coverage of PSMs within the specific range of PSM ARE, thus implying better PSM-level quantitation accuracy. A valid PSM is evaluated only if it is not a shared peptide and has no missing reporter ion.

Protein-level quantitation accuracy was also evaluated even though spectral library searching is originally used to enhance PSM identification, not protein-level quantitation. There are various protein ratio calculation algorithms, such as MedianPsmRatio, WeightedPsmRatio, and SumPsmIntensity^13^. Optimization of protein-level quantitation is beyond the scope of this study. Thus, we simply used SumPsmIntensity algorithm to demonstrate the quantitation performance at protein level. The algorithm is based on the summation of the reporter ion intensity of each channel across all the PSMs assigned to the protein. Protein ARE is used to evaluate the accuracy of quantitation at the protein level. Only proteins satisfying the 1% FDR cutoff at the protein level validation are evaluated.

## Results

### Determination of FPF conditions

FPF is a rule-based filtering mechanism that relies on a set of predefined conditions, in terms of features and their cutoffs, to remove PSMs with larger quantitation errors. To determine the suitable conditions, we analyzed the frequency distribution of PSMs with larger quantitation errors in PSM groups associated with each spectral feature in DB + SL-exclusive PSMs (2259 PSMs of human proteins, including 564 PSMs with larger quantitation errors) of DS-Schmidt, where the frequency of PSMs with larger quantitation errors (called frequency for short) is the number of such PSMs divided by the number of PSMs having a specific feature value or range, as shown in Supplementary Fig. S1. As observed from the figure, some features such as charge state, peptide length, and IPepDist can be associated with a clear threshold for filtering PSMs, as the frequency of PSM groups below and above the threshold differ significantly. For example, 61.9% (13 out of 21) PSMs with a peptide length of at least 25 are PSMs with larger quantitation errors; in contrast, for peptides with a length of at most 24, the frequency is 24.6% (551 out of 2238), indicating that the peptide length 25 can be a suitable threshold. Such results suggest that using spectral features as filtering conditions can be a valid approach to eliminate PSMs with larger quantitation errors from quantitation analysis.

Judging from the frequency distributions of PSMs with larger quantitation errors shown in Supplementary Fig. S1, we determined the filtering conditions as follows: (1) precursor’s charge state ≥ 5, (2) precursor’s mass ≥ 4000 Da, (3) peptide length ≥ 25, (4) average reporter ion intensity < 10,000, (5) IPepDist ≥ 0.8, (6) IProtDist ≥ 0.6, (7) absolute mass difference ≥ 2 Da, (8) dot product < 0.4, and (9) F-value < 0.4. A PSM is filtered out if any of the above-mentioned conditions is satisfied. These filtering conditions are in consistence with previous studies, for example, charge state and peptide length are reported to be positively correlated with quantitation error^22^. Longer peptides in general have larger precursor’s mass. It is also known that smaller reporter ion intensities lead to less quantitation accuracy^33–35^. Moreover, larger IPepDist and IProtDist indicate the PSM has larger ratio differences to other PSMs belonging to the same peptide and protein, respectively, implying that the PSM is likely to be an outlier and less quantitatively accurate. On the other hand, PSMs with larger absolute mass difference, smaller dot product, and smaller F-value are associated with less confident identifications or perhaps false positive hits, which are also likely to produce larger quantitation errors. Some features are not used because they do not reveal a clear threshold.

### Quantitation results on DS-schmidt at the PSM level

**Figure 2:**
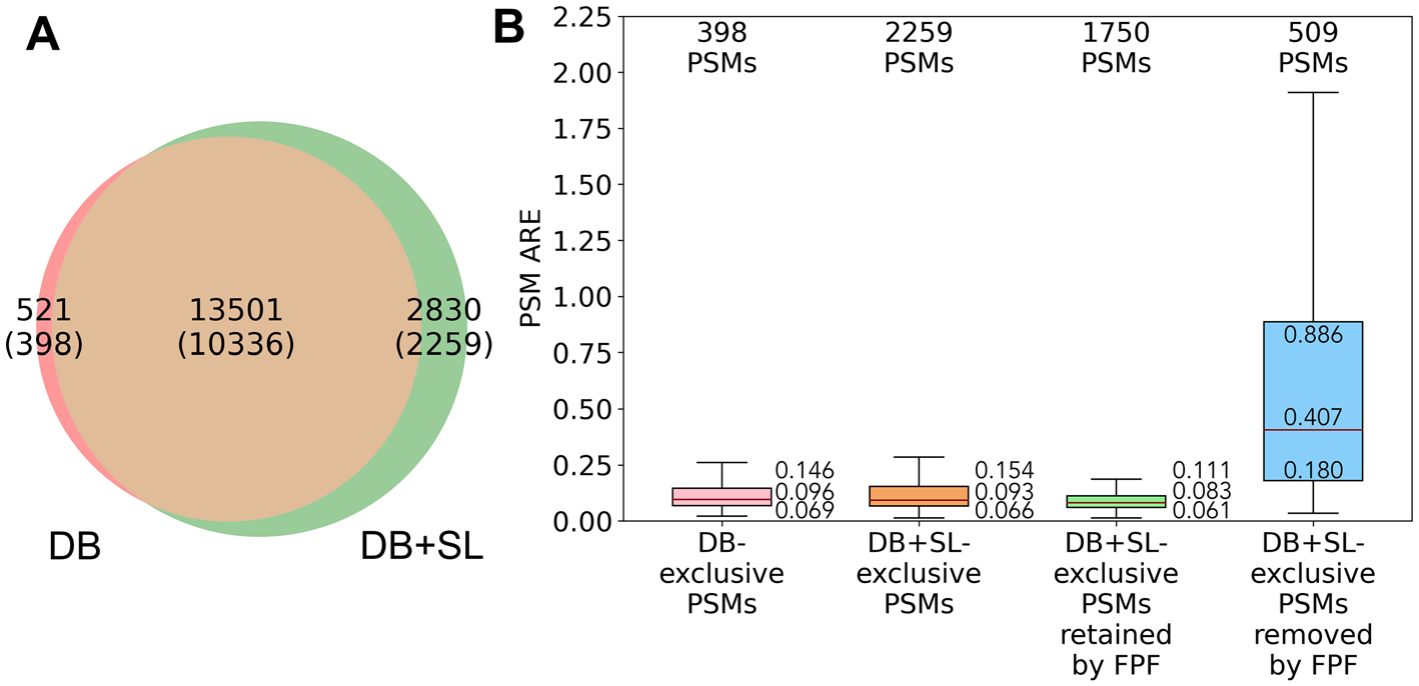
Analysis on valid PSMs of DB searching and DB + SL searching results of the DS-Schmidt data set. (**A**) Venn diagram of PSMs identified by DB searching and DB + SL searching. There are 398 of 521 DB-exclusive PSMs and 2259 of 2830 DB + SL-exclusive PSMs belonging to human proteins. (**B**) Distribution of PSM ARE for DB-exclusive PSMs, DB + SL-exclusive PSMs, DB + SL-exclusive PSMs retained by FPF, and DB + SL-exclusive PSMs removed by FPF. The three values beside each boxplot (from top to bottom) represent Q3, median, and Q1, respectively.

For DS-Schmidt, as explained in Materials and methods, we use the human proteins with known quantitation ratios to evaluate whether DB + SL searching combined with FPF improves quantitation accuracy compared to using DB searching alone. As shown in Fig. 2A, there are 521 DB-exclusive PSMs and 2830 DB + SL-exclusive PSMs, among which 398 DB-exclusive PSMs and 2259 DB + SL-exclusive PSMs belong to human proteins. Applying FPF on the 2259 DB + SL-exclusive PSMs removes 509 PSMs and retains 1750 PSMs. Figure 2B shows that the 398 DB-exclusive PSMs (pink boxplot) and 2259 DB + SL-exclusive PSMs (orange boxplot) have rather similar ARE distributions. The 509 DB + SL-exclusive PSMs removed by FPF (blue boxplot) have a significantly larger median PSM ARE of 0.407, whereas the 1750 DB + SL-exclusive PSMs retained by FPF (green boxplot) have a much smaller median PSM ARE of 0.083. The results demonstrate that FPF is capable of retaining PSMs of high quantitation accuracy while excluding those of low accuracy.

In this data set, a majority of DB + SL-exclusive PSMs have low quantitation errors, yet some of them have large ARE, as shown in Supplementary Fig. S2. For example, 321 DB + SL-exclusive PSMs have their AREs above 0.3, yet the number of DB-exclusive PSMs in this range is only 38, much less than DB + SL-exclusive PSMs. Such DB + SL-exclusive PSMs can deteriorate the quantitation accuracy of some proteins, rendering worse ARE than using DB searching alone. When FPF is applied on DB + SL-exclusive PSMs (green bars), a large number of PSMs with larger quantitation errors are removed while still retaining most PSMs with low quantitation errors. For example, 308 out of 321 PSMs with ARE above 0.3 are removed by FPF, whereas 1175 out of 1249 PSMs with ARE smaller than 0.1 are retained. Furthermore, DB + SL-exclusive PSMs retained by FPF has an AUC of 0.912 and compares favorably over the AUC of 0.855 and 0.819 for DB-exclusive PSMs and DB + SL-exclusive PSMs, respectively, as shown in Supplementary Fig. S3. The results show that through incorporating DB + SL searching with FPF, termed DB + SL + FPF, we include more PSMs with lower AREs and exclude PSMs with higher AREs from DB + SL-exclusive PSMs, thus improving overall quantitation accuracy.

### Quantitation results on DS-schmidt at the protein level

**Figure 3:**
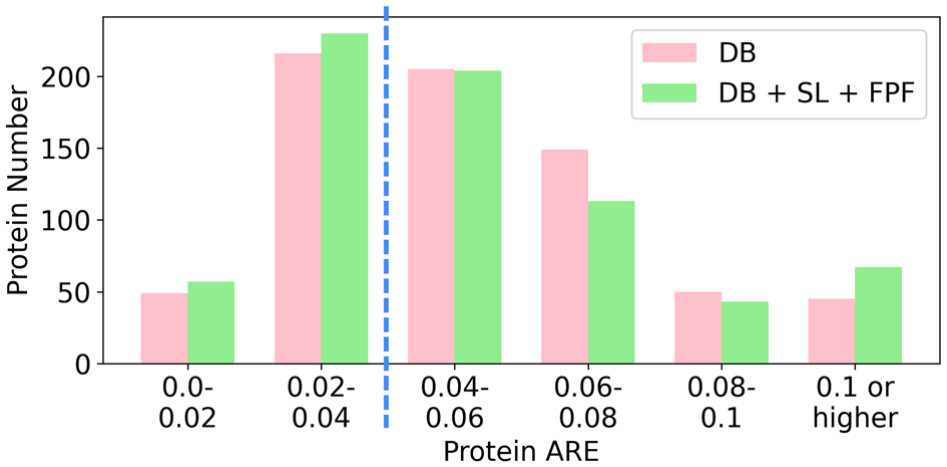
The number of proteins within different ranges of protein ARE for quantitation based on DB searching alone (pink bars) and that based on DB + SL + FPF (green bars) for DS-Schmidt.

For DS-Schmidt, the 398 DB-exclusive PSMs and 1750 DB + SL-exclusive PSMs retained by FPF can render different protein-level quantitation results for a total of 714 proteins, which account for 37.9% of the 1883 proteins identified by DB searching alone. For these proteins, we examined the distribution of protein numbers with different ranges of protein ARE for quantitation based on DB searching alone and that based on the proposed DB + SL + FPF. As shown in Fig. 3, the number of proteins with lower AREs increases, and that with higher AREs decreases for quantitation based on DB + SL + FPF. For example, the number of proteins with ARE < 0.04 increases from 265 to 287 (8.3% increase), and the number of proteins with ARE ≥ 0.04 decreases from 449 to 427 (4.9% decrease). Nevertheless, we also observe that the number of proteins with ARE ≥ 0.1 is larger for DB + SL + FPF because a small number of DB + SL-exclusive PSMs with larger quantitation errors cannot be completely removed by FPF, as shown in Fig. S2 of the Supporting Information.

The 714 proteins were further categorized by their abundances, which was calculated based on the average of reporter ion intensities across all the PSMs belonging to a protein. Supplementary Fig. S4 illustrates the protein level quantitation results for proteins of different abundances. For proteins with the bottom 25% abundances (Fig. S4A), quantitation based on DB + SL + FPF yields more proteins of larger ARE (ARE ≥ 0.1) compared to quantitation based on DB searching alone. In contrast, for proteins with the top 25% abundances (Fig. S4C), quantitation based on DB + SL + FPF yields more proteins of smaller ARE (ARE < 0.04) compared to quantitation based on DB searching alone. This is because proteins of higher abundances are associated with PSMs of larger reporter ion intensities and better signal quality, which benefits spectral library searching in finding other PSMs of good signal quality and high quantitation accuracy.

### PSM-level quantitation results on DS-NCI-7 and DS-Yang

**Figure 4:**
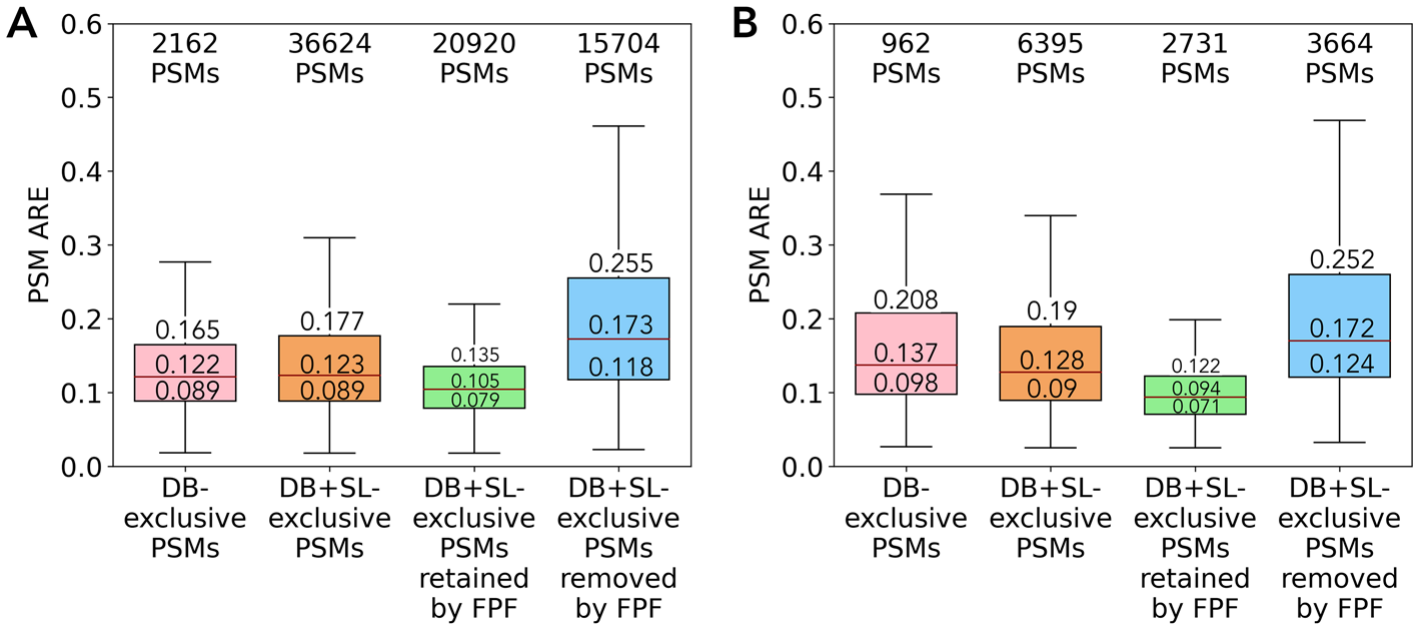
Distribution of PSM ARE for DB-exclusive PSMs, DB + SL-exclusive PSMs, DB + SL-exclusive PSMs retained by FPF, and DB + SL-exclusive PSMs removed by FPF on (**A**) PSMs in DS-NCI-7 and (**B**) PSMs in DS-Yang. The three values within each boxplot (from top to the bottom) represent Q3, median, and Q1, respectively.

As the filtering conditions of FPF are determined by DS-Schmidt, we evaluate the generalizability of the filter on DS-NCI-7 and DS-Yang at both PSM and protein levels. At the PSM level, for DS-NCI-7, in addition to 89,722 common PSMs identified by both DB searching and DB + SL searching, there are 2162 DB-exclusive PSMs and 36,624 DB + SL-exclusive PSMs, as shown in Supplementary Fig. S5, implying that the inclusion of SL searching significantly increases the identification coverage. As shown in Fig. 4A, the median ARE of DB-exclusive PSMs (pink boxplot) and DB + SL-exclusive PSMs (orange boxplot) are rather similar. Among the 36,624 DB + SL-exclusive PSMs, FPF filters out 15,704 PSMs with a median ARE of 0.173 (blue boxplot), significantly larger than the median ARE of 0.105 for the 20,920 remaining PSMs (green boxplot). It can also be observed that the 20,920 DB + SL-exclusive PSMs retained by FPF have a median PSM ARE of 0.105, smaller than the median PSM ARE of 0.122 for the 2162 DB-exclusive PSMs. The observation implies that DB + SL + FPF contains more accurate PSMs for quantitation compared to using DB searching alone. The distribution of PSM count across different PSM ARE ranges is illustrated in Supplementary Fig. S6, where the number of DB + SL-exclusive PSMs (orange bars), compared to that of DB-exclusive PSMs (pink bars), shows a drastic increase across both lower ARE ranges (e.g., ARE < 0.2) and higher ARE ranges (e.g., ARE > 0.3). Applying FPF on DB + SL-exclusive PSMs (green bars) effectively reduces the latter while retaining the majority of the former. Furthermore, the DB + SL-exclusive PSMs retained by FPF has an AUC of 0.894, compared favorably over the AUC of 0.857 and 0.84 for DB-exclusive PSMs and DB + SL-exclusive PSMs, respectively, as shown in Supplementary Fig. S7. The above phenomena are consistent with the observations from DS-Schmidt, even though the filtering conditions are determined by the analyses of DS-Schmidt instead of DS-NCI-7.

For DS-Yang, there are 980 DB-exclusive PSMs and 6574 DB + SL-exclusive PSMs, among which 962 DB-exclusive PSMs and 6395 DB + SL-exclusive PSMs belong to *E*. *coli* proteins, as shown in Supplementary Fig. S8, and are used for performance evaluation. DB-exclusive PSMs (pink boxplot) have marginally higher AREs than DB + SL-exclusive PSMs (orange boxplot), as shown in Fig. 4B. FPF filters out 3664 PSMs with a median ARE of 0.172 (blue boxplot), significantly larger than the median ARE of 0.094 for the 2731 remaining PSMs (green boxplot). The Q3, median, and Q1 AREs of 2731 DB + SL-exclusive PSMs retained by FPF are 0.122, 0.094, and 0.071, respectively, considerably smaller than 0.208, 0.137, and 0.098 for DB-exclusive PSMs. The situation indicates that more quantitatively accurate PSMs are in place of less accurate PSMs when using DB + SL + FPF for quantitation. The distribution of PSM count across different PSM ARE ranges shown in Supplementary Fig. S9 reveals a similar trend to DS-Schmidt and DS-NCI-7, namely, applying FPF on DB + SL-exclusive PSMs effectively reduces PSMs of higher AREs while retaining the majority of the PSMs of lower AREs. Furthermore, DB + SL-exclusive PSMs retained by FPF have an AUC of 0.905, which compares favorably over the AUC of 0.809 and 0.826 for DB-exclusive PSMs and DB + SL-exclusive PSMs, respectively, as shown in Supplementary Fig. S10.

### Quantitation results of DS-NCI-7 and DS-Yang at the protein level

**Figure 5:**
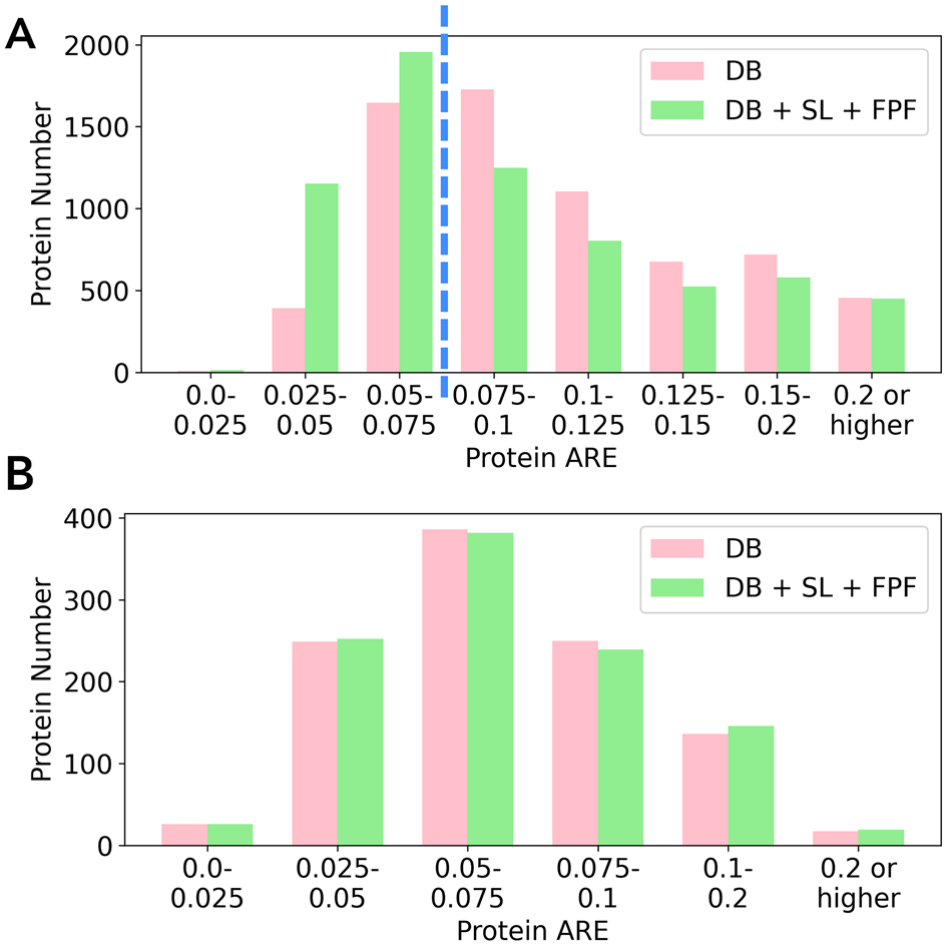
The number of proteins within different protein ARE ranges for quantitation based on DB searching alone and that based on DB + SL + FPF on (**A**) Human proteins in DS-NCI-7 and (**B**) *E. coli* proteins in DS-Yang.

For DS-NCI-7, a total of 2162 DB-exclusive PSMs and 20,920 DB + SL-exclusive PSMs retained by FPF correspond to 6727 human proteins (accounting for 74.7% of the 8998 proteins identified by DB searching), for which the protein-level quantitation results can be altered. For these proteins, the distributions of protein numbers with different ranges of protein ARE for quantitation based on DB searching alone and that based on DB + SL + FPF are shown in Fig. 5A. Quantitation based on DB + SL + FPF demonstrates a significant increase in the number of proteins with lower AREs and a significant decrease in the number of proteins with higher AREs. For example, the number of proteins with ARE < 0.075 increases from 2036 to 3114 (52.9% increase), whereas the number of proteins with ARE ≥ 0.075 decreases from 4691 to 3613 (23% decrease). The results reveal the generalizability of the FPF conditions though determined by a single large data set.

For DS-Yang, a total of 962 DB-exclusive PSMs and 2731 DB + SL-exclusive PSMs retained by FPF correspond to 1064 *E. coli* proteins, accounting for 46.0% of the 2314 *E. coli* proteins identified by DB searching. For these proteins, the distributions of protein numbers with different ranges of protein ARE for quantitation based on DB searching alone and that based on DB + SL + FPF are shown in Fig. 5B. Unlike the other two data sets which show considerable protein-level improvement for quantitation based on DB + SL + FPF, there are only marginal differences in the number of proteins across different protein ARE ranges. The outcome can be explained by the fact that the 962 DB-exclusive PSMs, and the 2731 DB + SL-exclusive PSMs retained by FPF and even the 6395 DB + SL-exclusive PSMs before filtering, are far less than the 51,487 common PSMs between the identification results of DB searching and DB + SL searching. In other words, both DB searching and DB + SL + FPF use similar sets of PSMs to quantify proteins, irrelevant of the filtering, thus producing similar protein quantitation outcomes. The scenario suggests that in cases where spectral library searching does not yield a substantial increase in PSM identification, namely, there being relatively few DB + SL-exclusive PSMs, the proposed pipeline may exhibit protein-level quantitation accuracy comparable to that of the conventional approach using DB searching alone.

### Case studies for exploring the efficacy of DB + SL + FPF

To understand why SL searching and FPF help improve protein quantitation accuracy, we particularly selected four proteins from the DS-Schmidt and DS-NCI-7 data sets for exploration, as summarized in Supplementary Table S5.

#### Case study of RBP56_HUMAN

As shown in Supplementary Table S5, the protein sp|Q92804|RBP56_HUMAN in DS-Schmidt has a protein ARE of 0.0789 for the quantitation based on DB searching alone. DB + SL searching yields 9 DB + SL-exclusive PSMs (the rows in blue color in Table 1) mostly with lower PSM ARE (7 out of 9 have a PSM ARE < 0.15); thus, quantitation based on DB + SL searching yields a reduced protein ARE of 0.0193. Applying FPF on 9 DB + SL-exclusive PSMs filters out one PSM with a much higher ARE of 0.5535 (the row with red text in Table 1). As a result, quantitation based on the 18 PSMs obtained by DB + SL + FPF further improves the protein ARE to 0.0187. Spectral features of each PSM are shown in Supplementary Table S6, in which the PSM removed by FPF satisfies the following filtering conditions. Its average reporter ion intensity of 5912 is smaller than the cutoff of 10,000; its IPepDist of 1.4691 is greater than the cutoff of 0.8; its IProtDist of 1.3082 is greater than the cutoff of 0.6.

**Table 1:**
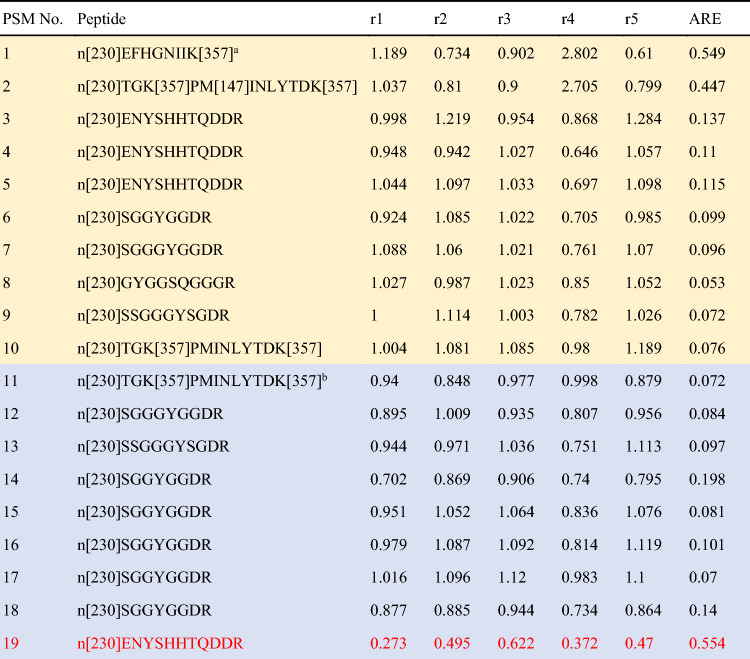
PSMs of the protein sp|Q92804|RBP56_HUMAN in DS-Schmidt.

^a^The rows in yellow color are common PSMs between the identification results of DB + SL searching and DB searching.

^b^Those in blue are DB + SL-exclusive PSMs, and the row with red text is DB + SL-exclusive PSMs removed by FPF.

#### Case study of MDC1_HUMAN

The protein sp|Q14676|MDC1_HUMAN in DS-Schmidt has a protein ARE of 0.1004 for quantitation based on DB searching alone (Supplementary Table S5). The protein has 9 DB + SL-exclusive PSMs, all of which pass the FPF filtering, as shown in Supplementary Table S7. The quantitation based on DB + SL searching improves the protein ARE to 0.0487. This case shows that the quantitation based on DB + SL searching can improve quantitation accuracy since the DB + SL-exclusive PSMs can be fairly accurate.

#### Case study of RL27A_HUMAN

The protein sp|P46776|RL27A_HUMAN in DS-Schmidt has a protein ARE of 0.1034 for quantitation based on DB searching alone (Supplementary Table S5). As shown in Supplementary Table S8, there is exactly one DB-exclusive PSM with a PSM ARE of 0.8579, and one DB + SL-exclusive PSM with a PSM ARE of 0.1827 passing the FPF filtering. Thus, the quantitation based on DB + SL searching leads to an improved protein ARE of 0.0211. The situation shows that combining SL and DB searching can exclude highly inaccurate DB-exclusive PSMs from quantitation, thus leading to improved quantitation.

#### Case study of NP_055874.2

The protein NP_055874.2 in DS-NCI-7 has a protein ARE of 0.1218 for quantitation based on DB searching alone (Supplementary Table S5). There are in total 76 DB + SL-exclusive PSMs, in which the 68 removed by FPF have an average PSM ARE of 0.567 and the remaining 8 have an average PSM ARE of 0.1172 (detailed spectral features are shown in Supplementary Table S9). This phenomenon explains why quantitation based on DB + SL searching generates a much larger protein ARE of 3.1154, and the quantitation based on DB + SL + FPF generates a much smaller protein ARE of 0.1001. This case demonstrates that though much-increased PSMs obtained by DB + SL searching can deteriorate protein ARE, applying FPF removes the majority of DB + SL-exclusive PSMs with larger quantitation errors and retains quantitatively accurate DB + SL-exclusive PSMs, thus yielding an improved protein ARE.

## Conclusions

Most of the existing isobaric-labeling quantitation relies on the identification results from DB searching. Identification through SL searching has been shown to provide higher sensitivity, yet its potential for quantitation remains largely unexplored. In this study, we propose a novel quantitation pipeline that combines DB searching, SL searching, and FPF to improve quantitation accuracy. FPF is a publicly available installation-free software tool that filters out noisy and quantitatively unreliable PSMs based on a discriminating set of spectral features. Our results on three different data sets demonstrate that the PSMs retained by FPF have significantly smaller average ARE than those removed by FPF. Taking DS-Schmidt as an example, the median PSM ARE for DB + SL-exclusive PSMs retained by FPF is 0.083, whereas that for DB + SL-exclusive PSMs removed by FPF is 0.407. We also show that the improved quantitation accuracy at the PSM level can propagate to the protein level, i.e., the number of proteins with lower ARE increases. Compared to protein quantitation based on DB searching, the quantitation based on DB + SL + FPF results in an 8.3% increase in the number of proteins with ARE < 0.04 for DS-Schmidt, and a 52.9% increase in the number of proteins with ARE < 0.075 for DS-NCI-7. This study shows that, in addition to its better sensitivity, SL searching can be used to improve quantitation in isobaric labeling experiments, incorporating with the usage of FPF. However, we also observed two possible limitations of the proposed DB + SL + FPF pipeline. First, a small number of proteins may yield increased ARE because of FPF unable to completely remove their PSMs with larger quantitation errors. Second, if applying DB + SL searching to a data set does not substantially increase the number of PSMs compared to DB searching alone, the data set may exhibit limited overall quantitation improvement using our pipeline. Nevertheless, the proposed DB + SL + FPF pipeline generally shows higher quantitation accuracy than the conventional approach based on DB-searching results alone.

### Supplementary Information

Supplementary Information 1.

Supplementary Information 2.

## Data Availability

FPF executable files, sample data sets, and user manual are freely available at https://ms.iis.sinica.edu.tw/comics/Software_FPF.html. The MS data sets analyzed during the current study are public data available in the ProteomeXchange Consortium via the PRIDE repository with identifiers PXD003346 and PXD005486, and in CPTAC Data Portal with identifier PDC000295.
