# Supplementary material for: Improving quantitation accuracy in isobaric-labeling mass spectrometry experiments with spectral library searching and feature-based peptide-spectrum match filter
Source: Sci Rep. 2023 Aug 29;13:14119. doi: 10.1038/s41598-023-41124-2 (PMC10465558; doi:10.1038/s41598-023-41124-2)
Supplement: Supplementary file 1 — Supplementary Information 1. [file 41598_2023_41124_MOESM1_ESM.pdf]

Supplementary Information

Improving quantitation accuracy in isobaric-labeling mass spectrometry experiments with spectral library searching and feature-based peptide-spectrum match filter

Tzu-Yun Kuo[1], Jen-Hung Wang[2,3,4], Yung-Wen Huang[5], Ting-Yi Sung[3,*], and Ching-Tai Chen[6,7,*]

1 Department of Biochemical Science and Technology, College of Life Science, National Taiwan University, Taipei 10617, Taiwan

2 Bioinformatics Program, Taiwan International Graduate Program, Institute of Statistical Science, Academia Sinica, Taipei 11529, Taiwan

3 Institute of Information Science, Academia Sinica, Taipei 11529, Taiwan

4 Institute of Biomedical Informatics, National Yang Ming Chiao Tung University, Taipei 11221, Taiwan

5 Department of Computer Science and Information Engineering, National Taiwan University, Taipei 10617, Taiwan

6 Department of Bioinformatics and Biomedical Engineering, Asia University, Taichung 41354, Taiwan

7 Center for Precision Health Research, Asia University, Taichung 41354, Taiwan

*Correspondence: tsung@iis.sinica.edu.tw, ctchen@asia.edu.tw

Table S1. Parameters for database (DB) searching with Comet and X!Tandem.

**Parameter**	**DS-Schmidt**	**DS-NCI-7**	**DS-Yang**
Database	20625 *Homo sapiens*, 1466 *Bartonella henselae*, 6 calibration mixed proteins, and 381 commonly observed contaminants	55788 *Homo sapiens*	4149 *E. coli* and 13 spiked proteins
Isobaric labeling tag	TMT-6	TMT-10	TMT-10
Precursor ion mass tolerance	10 ppm	20 ppm	20 ppm
Fragment ion mass tolerance	0.02 Da	0.06 Da	0.02 Da
Search enzyme	Fully-digested trypsin	Fully-digested trypsin	Fully-digested trypsin
Maximum number of cleavages	2	2	3
Fixed modifications	Carbamidomethylation (C), TMT6plex (K and peptide n-terminus)	Carbamidomethylation (C), TMT6plex (K and peptide n-terminus)	Carbamidomethylation (C), TMT6plex (K and peptide n-terminus)
Variable modifications	Oxidation (M)	Oxidation (M), Phosphorylation (S, T, Y)	Oxidation (M), Deamidation (N, Q)

Table S2. Parameters for PeptideProphet.

**Command-line Token**	**Parameter details**	**Parameter value**
-p<prob>	Results below this PeptideProphet probability are filtered out.	0.05
-l<num>	Minimum peptide length considered	7
-PPM	Accurate mass binning with PPM as unit	on
-OAPd	Using decoy hits to pin down the negative distribution	on
-d	Decoy prefix	'REV_' for DB search of DS-Schmidt; Otherwise 'DECOY_'

Table S3. Parameters for spectral library (SL) construction.

**Command-line Token**	**Parameter name**	**Parameter value**
Library import options		
-cP<prob>	minimumProbabilityToInclude	DS-Schmidt: 0.6<br>DS-NCI-7: 0.55<br>DS-Yang: 0.7
Consensus library (applicable with the -cAC option)		
-cJ	combineAction	-cJU (union)
-cr<num>	minimumNumReplicates	1
Quality filter options (applicable with the -cAQ option)		
-cr<num>	minimumNumReplicates	1
-cL<level>	qualityLevelRemove	2
-cl<level>	qualityLevelMark	5
Decoy generation options (applicable with the -cAD option)		
-cc	decoyConcatenate	on
-cy<num>	decoySizeRatio	1

Table S4. Parameters for SL searching.

**Command-line Token**	**Parameter name**	**Parameter value**
-sM<tol>	precursorMzTolerance	0.5
-s_TMT	filterTMTReporterPeaks	on
-sE<ext>	outputExtension	-sEpep.xml

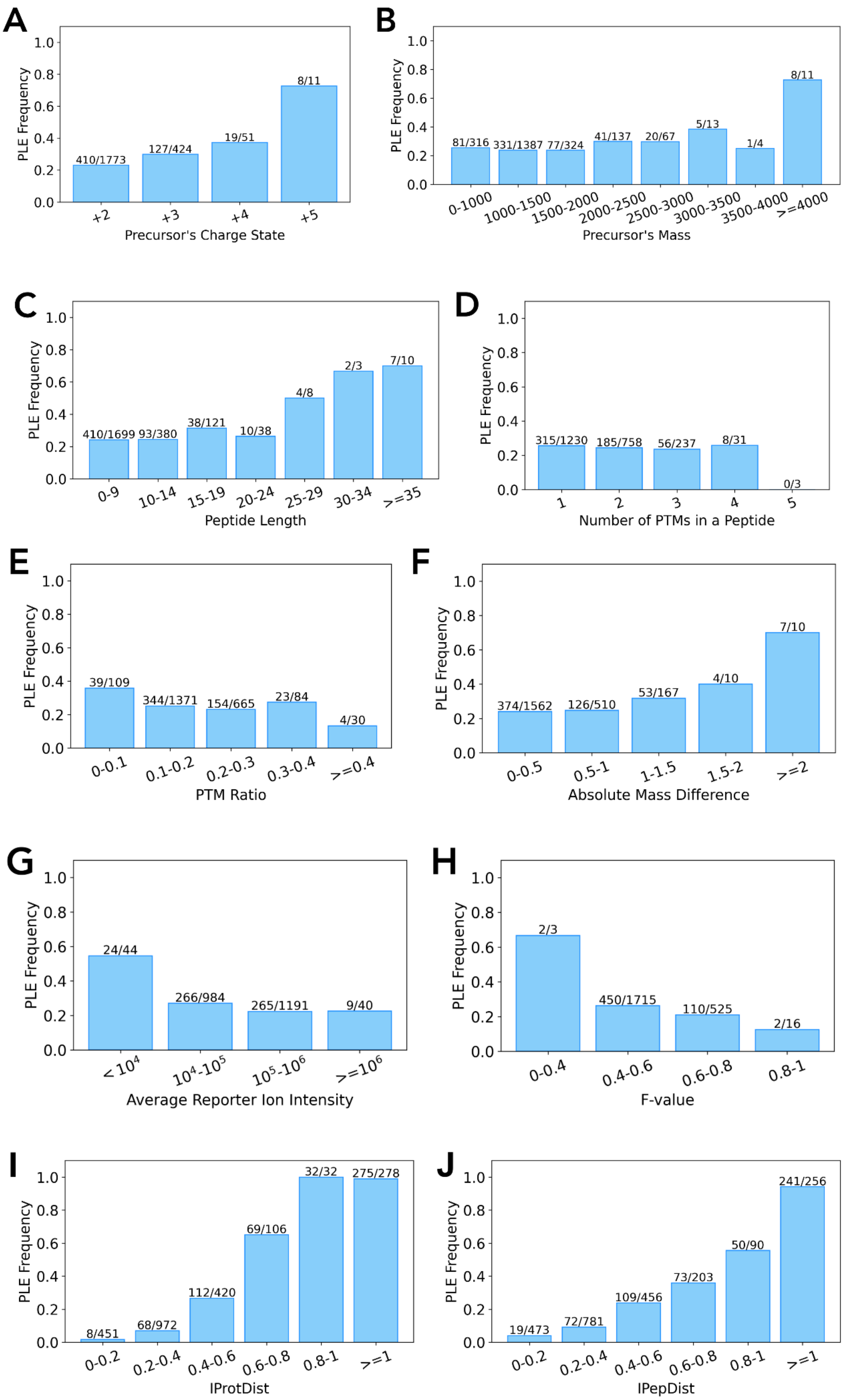
A
PLE Frequency
410/1773
127/424
19/51
8/11
+2
+3
+4
+5
Precursor's Charge State
B
PLE Frequency
81/316
331/1387
77/324
41/137
20/67
5/13
1/4
8/11
0-1000
1000-1500
1500-2000
2000-2500
2500-3000
3000-3500
3500-4000
>=4000
Precursor's Mass
C
PLE Frequency
410/1699
93/380
38/121
10/38
4/8
2/3
7/10
0-9
10-14
15-19
20-24
25-29
30-34
>=35
Peptide Length
D
PLE Frequency
315/1230
185/758
56/237
8/31
0/3
1
2
3
4
5
Number of PTMs in a Peptide
E
PLE Frequency
39/109
344/1371
154/665
23/84
4/30
0-0.1
0.1-0.2
0.2-0.3
0.3-0.4
>=0.4
PTM Ratio
F
PLE Frequency
374/1562
126/510
53/167
4/10
7/10
0-0.5
0.5-1
1-1.5
1.5-2
>=2
Absolute Mass Difference
G
PLE Frequency
24/44
266/984
265/1191
9/40
$<10^4$
10^4-10^5
10^5-10^6
$>=10^6$
Average Reporter Ion Intensity
H
PLE Frequency
2/3
450/1715
110/525
2/16
0-0.4
0.4-0.6
0.6-0.8
0.8-1
F-value
I
PLE Frequency
8/451
68/972
112/420
69/106
32/32
275/278
0-0.2
0.2-0.4
0.4-0.6
0.6-0.8
0.8-1
>=1
IProtDist
J
PLE Frequency
19/473
72/781
109/456
73/203
50/90
241/256
0-0.2
0.2-0.4
0.4-0.6
0.6-0.8
0.8-1
>=1
IPepDist

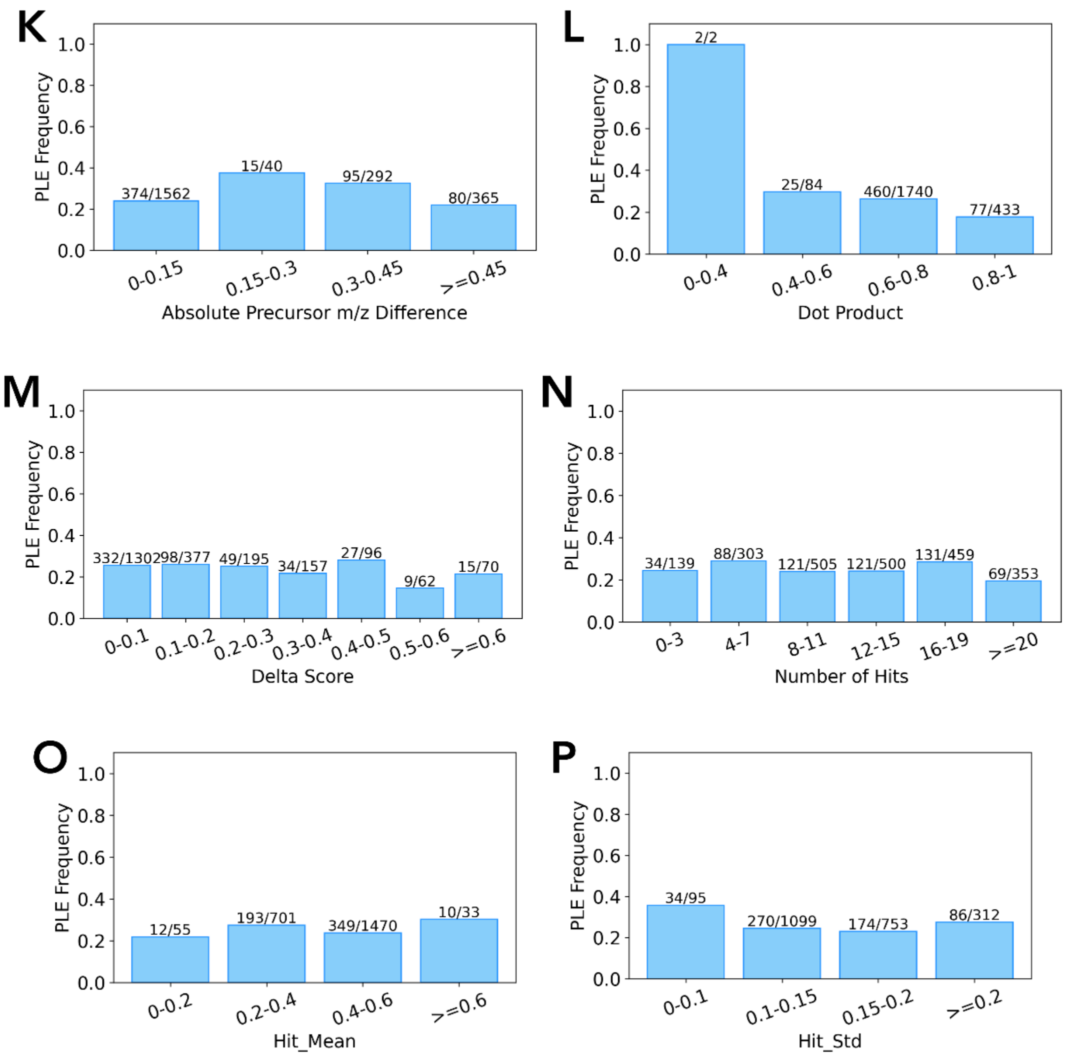

Figure S1. Frequency distributions of PSMs with larger quantitation errors in PSM groups associated with sixteen spectral features of DB+SL-exclusive PSMs in the DS-Schmidt data set. (A) precursor's charge state, (B) precursor's mass, (C) peptide length, (D) number of PTMs in a peptide, (E) PTM ratio, (F) absolute mass difference, (G) average reporter ion intensity, (H) F-value, (I) intra-protein distance (IProtDist), (J) intra-peptide distance (IPepDist), (K) absolute precursor m/z difference, (L) dot product, (M) delta score, (N) number of hits, (O) mean of the dot products of the hits (hit_mean), and (P) standard deviation of the dot products of the hits (hit_std).

The total number of DB+SL-exclusive PSMs is 2259, of which the 25% (564 PSMs) with the top AREs are regarded as PSMs with larger quantitation errors and denoted by PLEs. For a specific feature range, PLE frequency is defined as the number of PLEs divided by the number of DB+SL-exclusive PSMs.

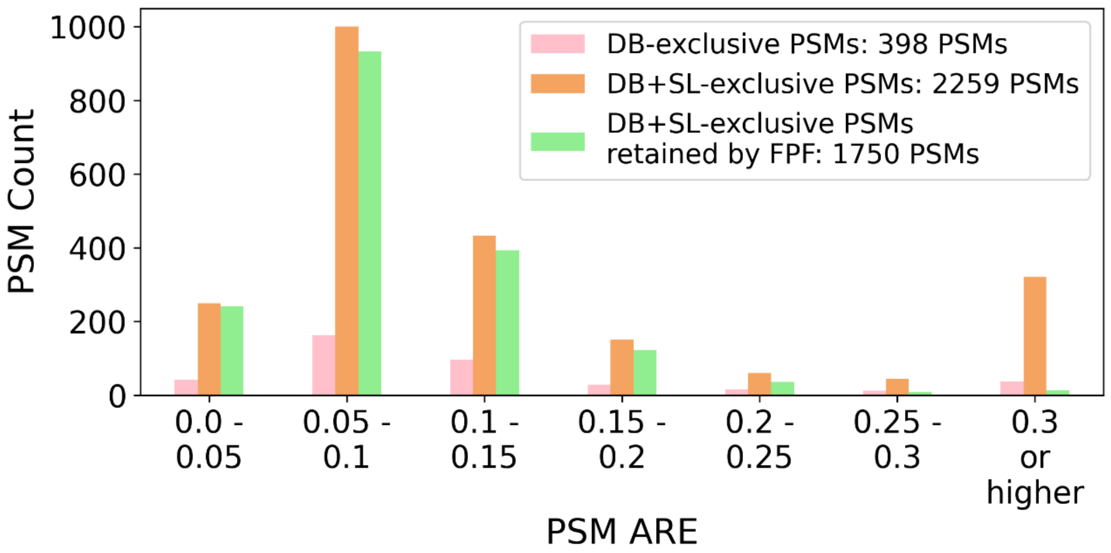

Figure S2. The number of PSMs within different PSM ARE range for DB-exclusive PSMs, DB+SL-exclusive PSMs, and DB+SL-exclusive PSMs retained by FPF for the human proteins of the DS-Schmidt data set. ARE: average relative error.

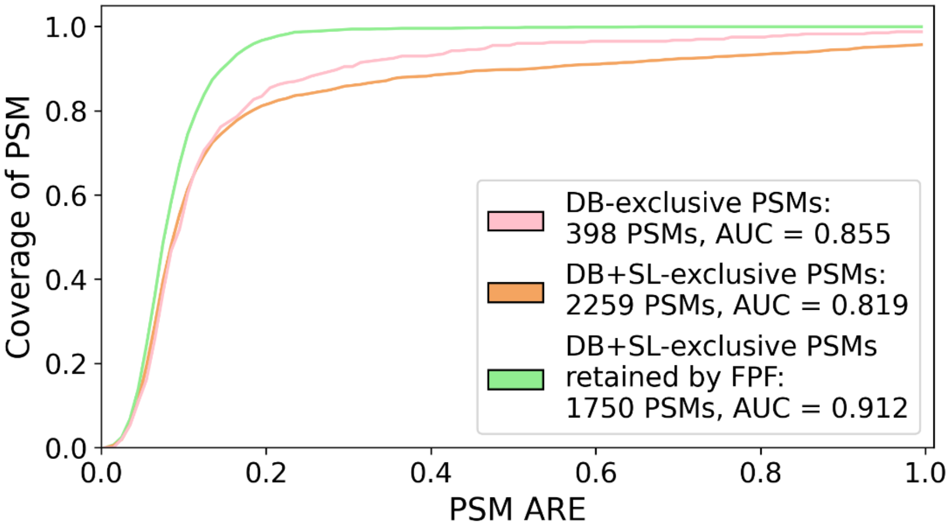

Figure S3. The curve of PSM coverage vs. PSM ARE for DB-exclusive PSMs, DB+SL-exclusive PSMs, and DB+SL-exclusive PSMs retained by FPF belonging to human proteins of the DS-Schmidt data set. AUC refers to the area under the curve of PSM coverage vs. PSM ARE. ARE: average relative error.

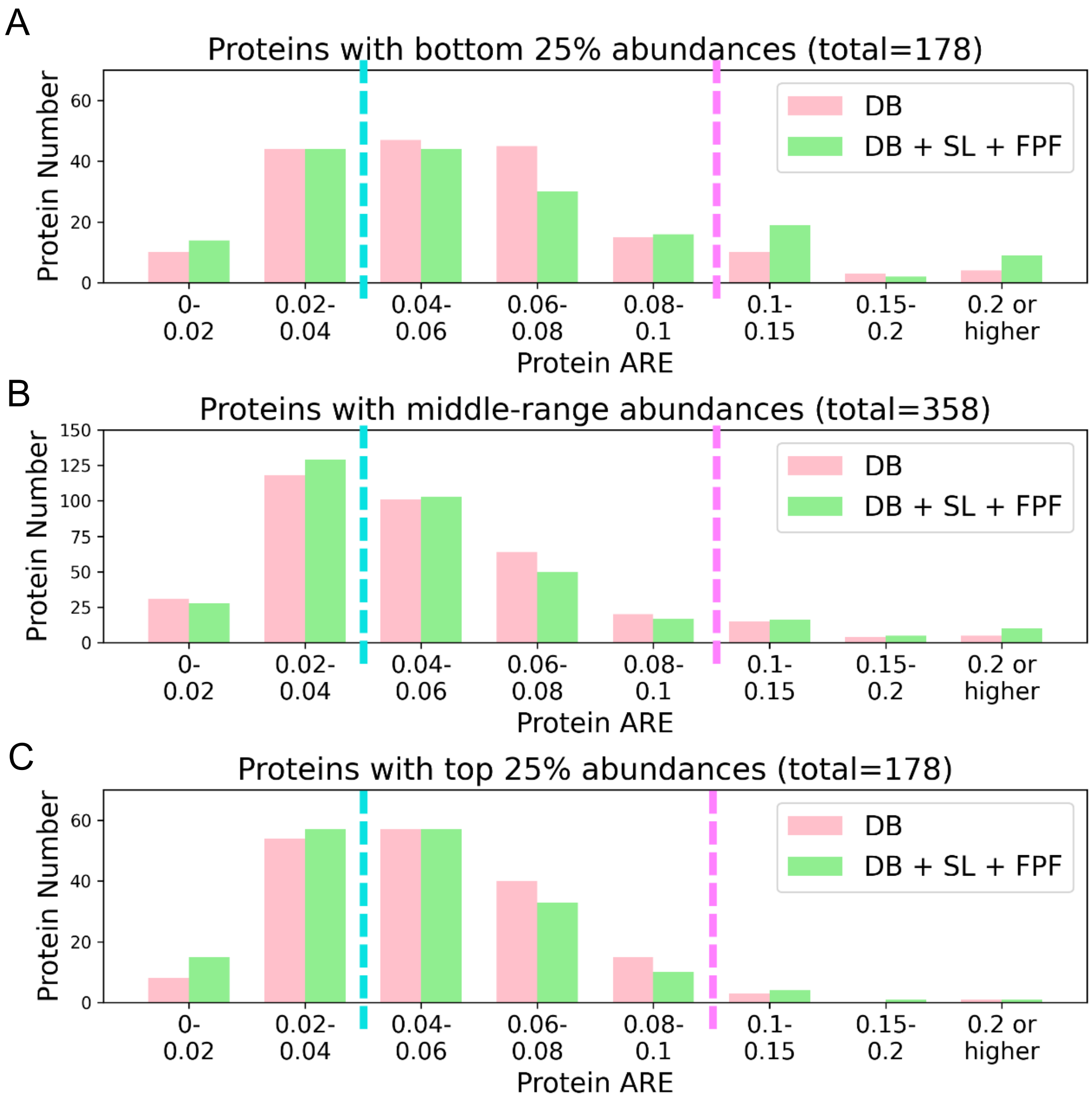

Figure S4. Protein-level quantitation analysis for proteins with (A) bottom 25% abundances, (B) middle-range abundances, and (C) top 25% abundances.

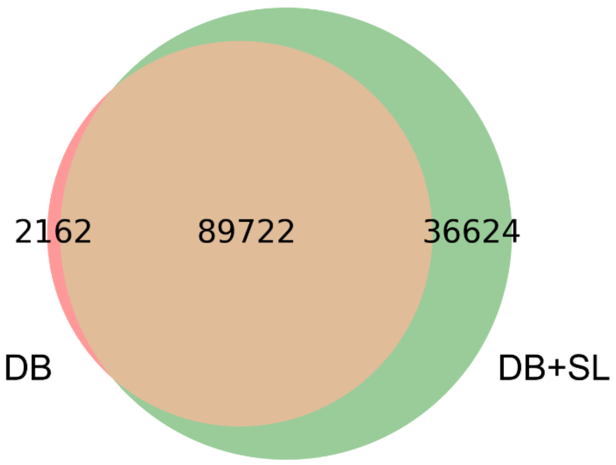

Figure S5. Venn diagram of PSMs identified by DB searching and DB+SL searching for the DS-NCI-7 data set.

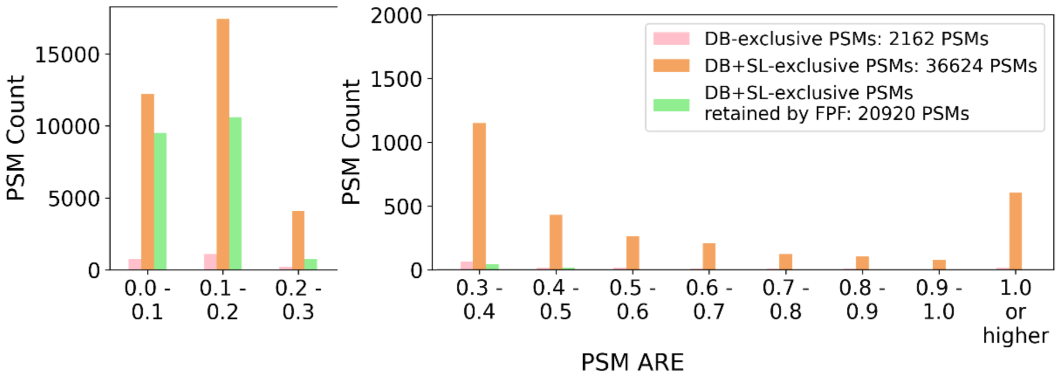

Figure S6. The number of PSMs within different PSM ARE range for DB-exclusive PSMs, DB+SL-exclusive PSMs, and DB+SL-exclusive PSMs retained by FPF for the DS-NCI-7 data set. ARE: average relative error.

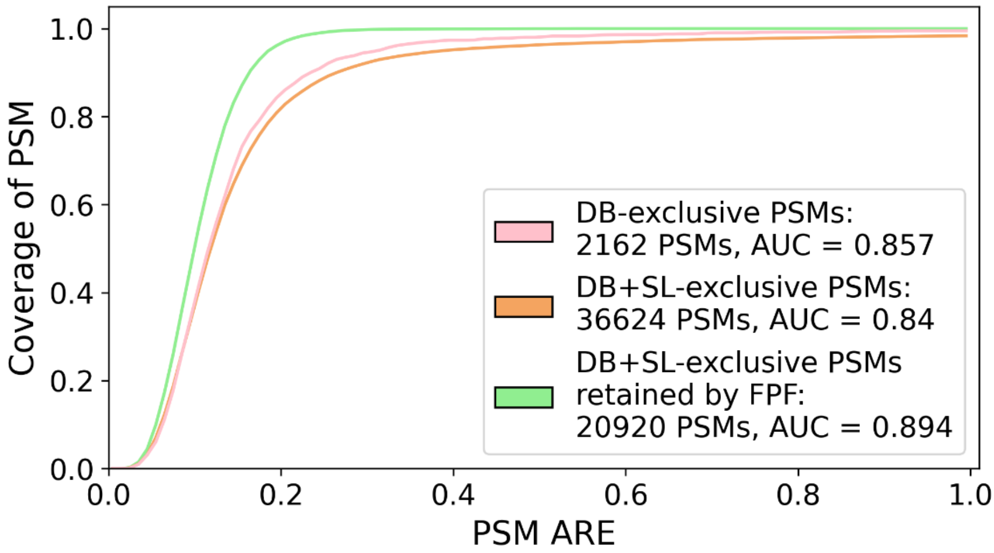

Figure S7. The curve of PSM coverage vs. PSM ARE for DB-exclusive PSMs, DB+SL-exclusive PSMs, and DB+SL-exclusive PSMs retained by FPF for the DS-NCI-7 data set. ARE: average relative error. AUC refers to the area under the curve of PSM coverage vs. PSM ARE.

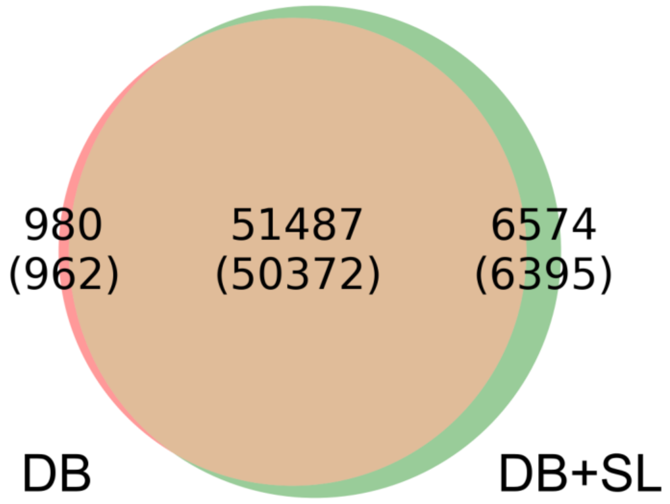

Figure S8. Venn diagram of PSMs identified by DB searching and DB+SL searching for the DS-Yang data set. 962 of 980 DB-exclusive PSMs and 6395 of 6574 DB+SL-exclusive PSMs belong to *E. Coli* proteins.

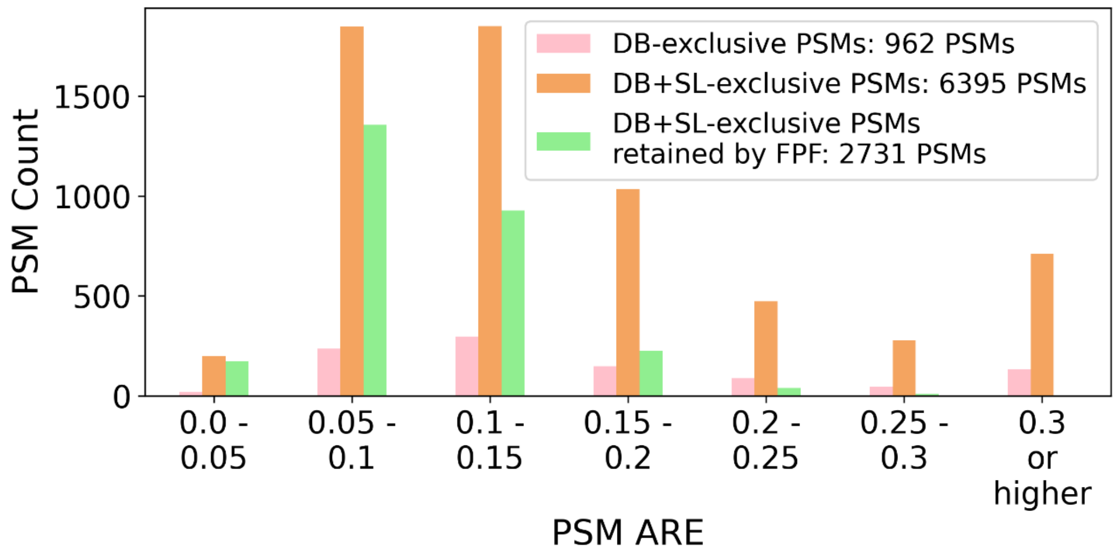

Figure S9. The number of PSMs within different PSM ARE range for DB-exclusive PSMs, DB+SL-exclusive PSMs, and DB+SL-exclusive PSMs retained by FPF for the *E. coli* proteins of the DS-Yang data set. ARE: average relative error.

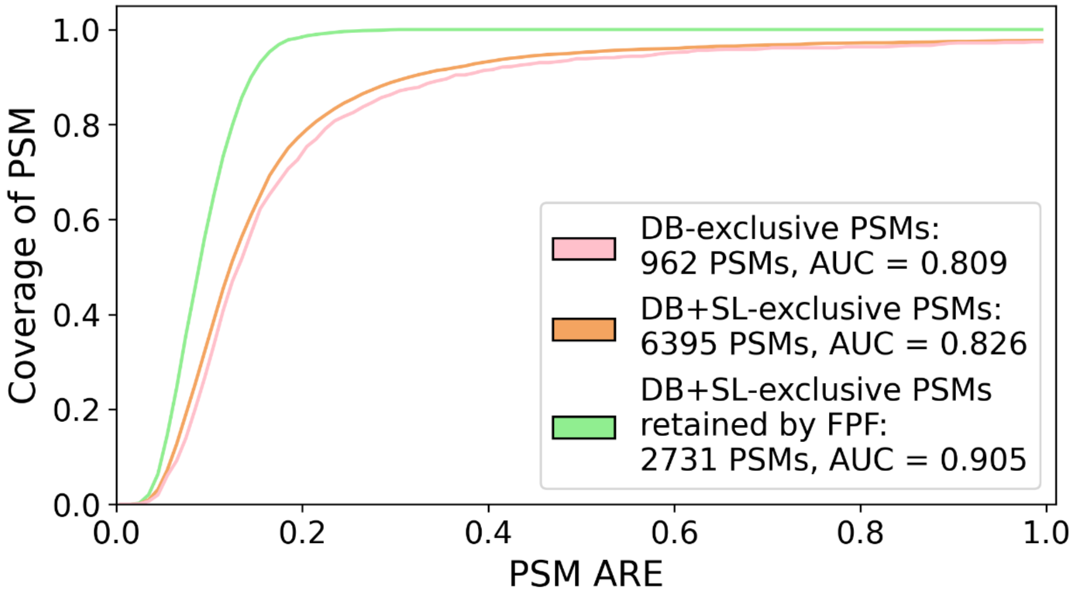

Figure S10. The curve of PSM coverage vs. PSM ARE for DB-exclusive PSMs, DB+SL-exclusive PSMs, and DB+SL-exclusive PSMs retained by FPF, corresponding to *E. coli* proteins of the DS-Yang data set. ARE: average relative error. AUC refers to the area under the curve of PSM coverage vs. PSM ARE.
